# Supplementary material for: Adaptive querying for reward learning from human feedback
Source: Front Robot AI. 2026 Feb 12;12:1734564. doi: 10.3389/frobt.2025.1734564 (PMC12935605; doi:10.3389/frobt.2025.1734564)
Supplement: Supplementary file 1 [file DataSheet1.pdf]

frontiers

Supplementary

This supplementary material presents additional simulation results, discusses the human subjects pilot study in simulation, and provides additional details on the in-person user study with a Kinova arm.

1 ADDITIONAL EXPERIMENTS IN SIMULATION

1.1 Effect of Clustering

To understand how clustering influences the effectiveness of our approach (AFS), we evaluate AFS under different clustering configurations. In particular, we vary the number of clusters and the clustering approach. Figure S1 shows the average penalty incurred using AFS with the KMeans and KCenters clustering algorithms for varying numbers of clusters ($K = \{2, 3\}$ in the navigation, vase and push domains). We restrict our evaluation to these K values since the maximum number of distinct clusters in each domain is determined by number of unique combinations of state features. In the navigation domain, features used for clustering states are $\langle f, p \rangle$. The valid unique combinations are $\langle f = \text{concrete}, p = \text{no puddle} \rangle$, $\langle f = \text{grass}, p = \text{no puddle} \rangle$, and $\langle f = \text{grass}, p = \text{puddle} \rangle$. Hence, having $K > 3$ will not produce unique clusters. Similarly, in the vase domain, features used for clustering are $\langle v, c \rangle$, where the unique, valid combinations are $\langle \text{no vase, no carpet} \rangle$, $\langle \text{vase, no carpet} \rangle$, $\langle \text{vase, carpet} \rangle$. For the push domain, the features used for clustering are $\langle b, w, h \rangle$, with valid unique combinations including $\langle \text{no box}, \text{not wrapped}, \text{hazard} \rangle$, $\langle \text{box}, \text{not wrapped}, \text{hazard} \rangle$, $\langle \text{no box}, \text{not wrapped}, \text{no hazard} \rangle$, and $\langle \text{box}, \text{wrapped}, \text{no hazard} \rangle$. The results in Figure S1 demonstrate that increasing K generally improves the performance of our approach, with both clustering methods. A higher number of clusters allows for a more refined grouping of states based on distinct state features, enabling the agent to query the human for feedback across a more diverse set of states. This diversity enhances the agent's ability to accurately learn and mitigate NSEs.

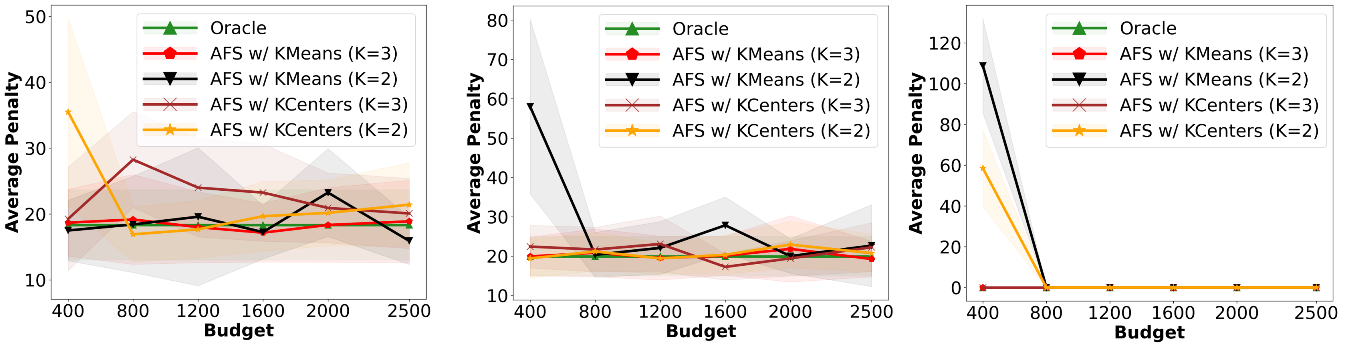

(a) Navigation: Unavoidable NSE (b) Vase: Unavoidable NSE (c) Safety-gym Push

Figure S1: Average penalty incurred using our approach (AFS) with KMeans and KCenters clustering algorithm, evaluated across varying number of clusters (K).

1.2 Learning with Implicit and Explicit Feedback Formats

To demonstrate AFS's flexibility across feedback modalities, we extend it to handle both explicit (e.g., DAM) and implicit (e.g., gaze) feedback formats.

1.2.1 Implicit Feedback Format

We consider a human's gaze on the screen as the implicit feedback format. Here, the robot requests to collect gaze data of the user and compares its action outcomes with the gaze positions of the user (Saran et al., 2021). Actions with outcomes aligning with the average gaze direction are labeled as acceptable (l_a), and unacceptable (l_h) otherwise.

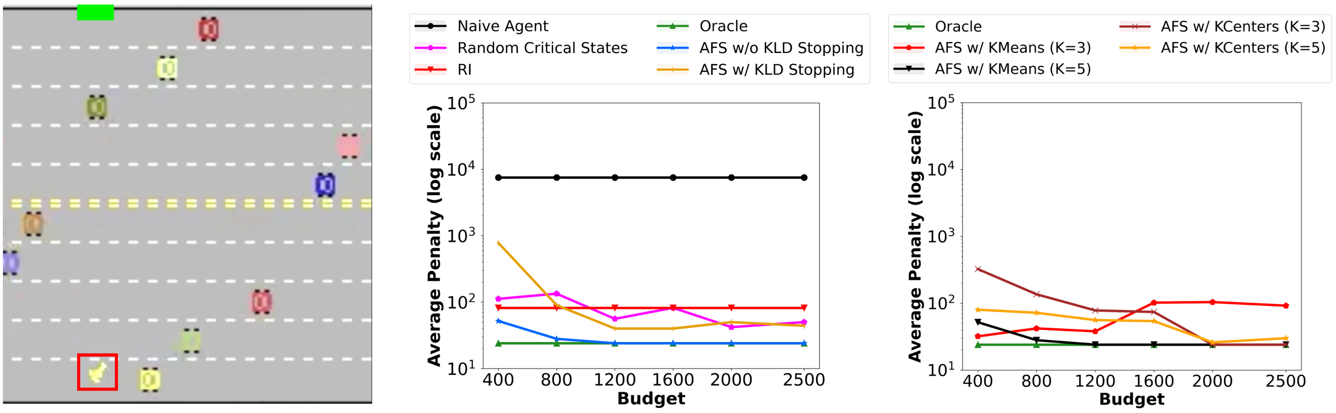

(a) ATARI Freeway: Avoidable NSE (b) Average penalty incurred (c) Average penalty incurred with KMeans and KCenters clustering.

Figure S2: An instance of the Freeway domain, and the average penalty incurred.

1.2.2 Domain

We evaluate AFS in the Atari Freeway environment, where the robot (a chicken) navigates ten cars moving at varying speeds to reach the destination quickly while avoiding being hit(Figure S2(a)). Being hit by a car moves the robot back to its previous position, and is a severe NSE. A game state is defined by coordinates (x_1, y_1) and (x_2, y_2), i.e., the top left and bottom right corners of the robot and cars, extracted from the Atari-HEAD dataset (Zhang et al., 2020). Similar to (Saran et al., 2021), only car coordinates within a specific range of the robot are considered. The robot can move up, down or stay in place, with unit cost and deterministic transitions.

1.2.3 Results and Discussion

Effect of Learning using AFS. Figure S2(b) shows the average NSE penalties when operating based on an NSE model learned using different querying approaches. Clusters for critical state selection were generated using KMeans clustering algorithm with $K = 5$ in the Atari Freeway domain. Table S1 shows the average cost for task completion. While the Naive Agent has a lower cost for task completion, it incurs the highest NSE penalty as it has no knowledge of R_N. RI causes more NSEs, as its reward function does not fully model the penalties for mild and severe NSEs. Overall, the results show that AFS consistently mitigates NSEs, without affecting the task performance substantially.

Table S1: Average cost at task completion.

Method	Avg. Cost
Oracle	3759.8 ± 0.00
Naive	61661.0 ± 0.00
RI	71716.6 ± 0.00
AFS (Ours)	1726.5 ± 0.00

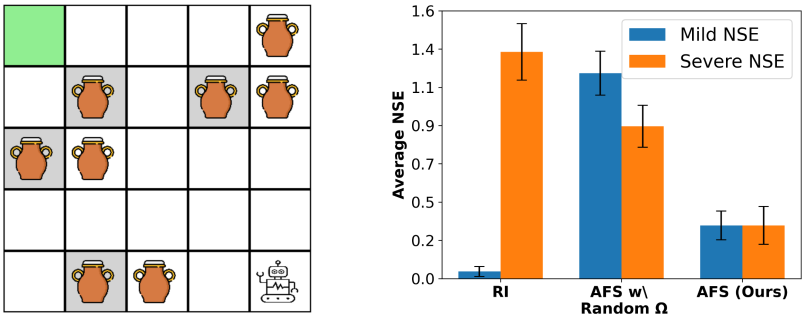

(a) Vase domain setup (b) Average number of NSEs

Figure S3: Results from the user study on a simulated domain.

2 HUMAN SUBJECTS PILOT STUDY IN SIMULATION

We conducted a within-subjects pilot study on a 5×5 Vase domain in simulation as shown in Fig. S3(a), with 12 human participants who had completed at least one course in Reinforcement Learning. The objective of this study is to evaluate whether: (1) AFS outperforms the baselines when a feedback preference model is learned from user interactions; (2) the selected feedback formats and critical states enhance agent's learning, and align with user preferences. The study was conducted with approval from Oregon State University IRB, and the participants were compensated with a $\$10$ Amazon gift card for their time.

2.1 Study Design

After introducing the domain and the agent's objective, users completed a tutorial where they interacted with the system by providing feedback in each of the six formats. The study interface included feedback buttons that varied based on the format. This was followed by a calibration phase, during which the users' preference model was learned. Each user was prompted five times per format to provide feedback, with the option to respond or ignore, allowing them to express their interaction preferences. The probability of receiving feedback in a given format was determined by the fraction of prompts the user responded to, while the cost was based on their self-reported effort.

The study comprised three phases, each evaluating a different baseline approaches to select feedback queries: (1) RI, (2) AFS with Random Ω, and (3) AFS with our proposed method for critical state selection. To prevent bias, users were unaware of the approach used in each phase. After completing a phase, they were shown a trajectory of the agent's learned policy and asked to evaluate the approach used in that phase.

2.2 User Interface for Feedback Collection

Figure S4 illustrates the interface used in the simulation-based human subjects study. Participants interacted with the simulated robot through a GUI consisting of feedback buttons whose labels and available options varied depending on the feedback format. For each query, the interface would display the action the agent intends to take in the gridworld and provide a corresponding set of input buttons to record user feedback.

This interface design allowed participants to provide both categorical and comparative feedback efficiently. After an initial training phase to practice providing feedback in different formats, participants self-reported the probability, $\psi(f)$, of providing feedback in a given format f, and effort ratings, $C(f)$.

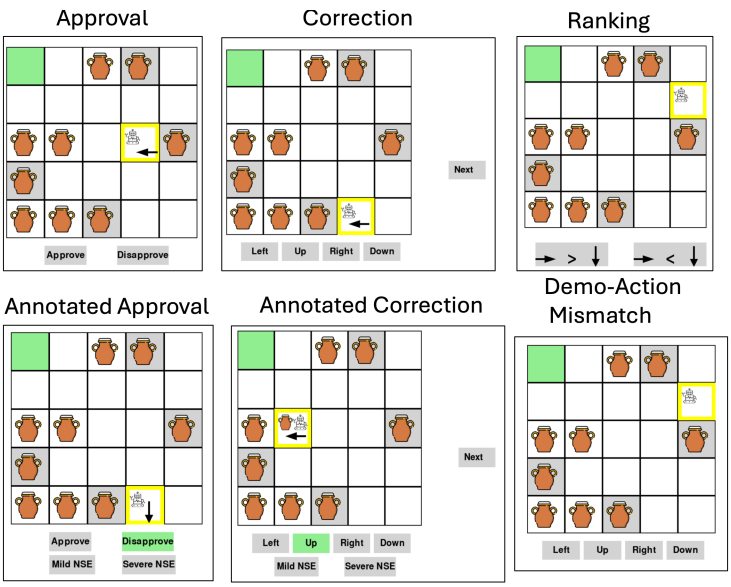

Figure S4: Interface for the human subjects study in simulation. Participants provide feedback via button clicks, with available options varying by format, as shown.

Table S2. Participants' qualitative assessment from the pilot study on a simulated domain.

Approach	Intelligent Feedback	Critical Points (%)			Improved Performance (%)	
		Yes	No	Overlap	Yes	No
RI	3.33 ± 1.23	83.30 ± 0.37	16.70 ± 0.37	73.47 ± 5.49	91.70 ± 0.28	8.30 ± 0.28
AFS w/ Random Ω	2.82 ± 0.94	66.70 ± 0.47	33.30 ± 0.94	75.51 ± 5.27	41.70 ± 0.49	58.30 ± 0.49
AFS (Ours)	3.25 ± 0.83	100.00 ± 0.00	0.00 ± 0.00	81.63 ± 4.94	100.00 ± 0.00	0.00 ± 0.00

2.3 Results and Takeaways

Fig. S3(b) shows that our approach tends to result in fewer NSEs, compared to the baselines. Since the NSE penalty is an aggregate measure that obscures severity distribution, we report exact NSE encounters by category for this study. Table S2 reports average over responses to our questions: "On a scale of 1 to 5, how intelligent do you think the agent's choice of feedback formats are, given your preferences?", "Were the states in which the agent requested for feedback critical to its learning?", and "Did the agent's performance improve at the end of the learning phase?". In addition, we also report the overlap between user-identified important query points and query points chosen by each approach.

Overall, the results of this pilot study indicate that (1) AFS tends to effectively select query points and lead to improved learning outcomes, when operating under a learned feedback model; and (2) AFS's performance in this pilot study where users interact with a simulated agent is comparable to that of our results in simulation. Building on these results and the insights gained from this pilot study, we next conduct a user study where human participants interact with a physical robot. Such a setting will enable us to evaluate how well the observed trends extend to human-robot physical interactions, and how that affects the usability, trust and the users' perceived workload when interacting with a system that learns using AFS.

3 IN-PERSON USER STUDY WITH KINOVA ARM

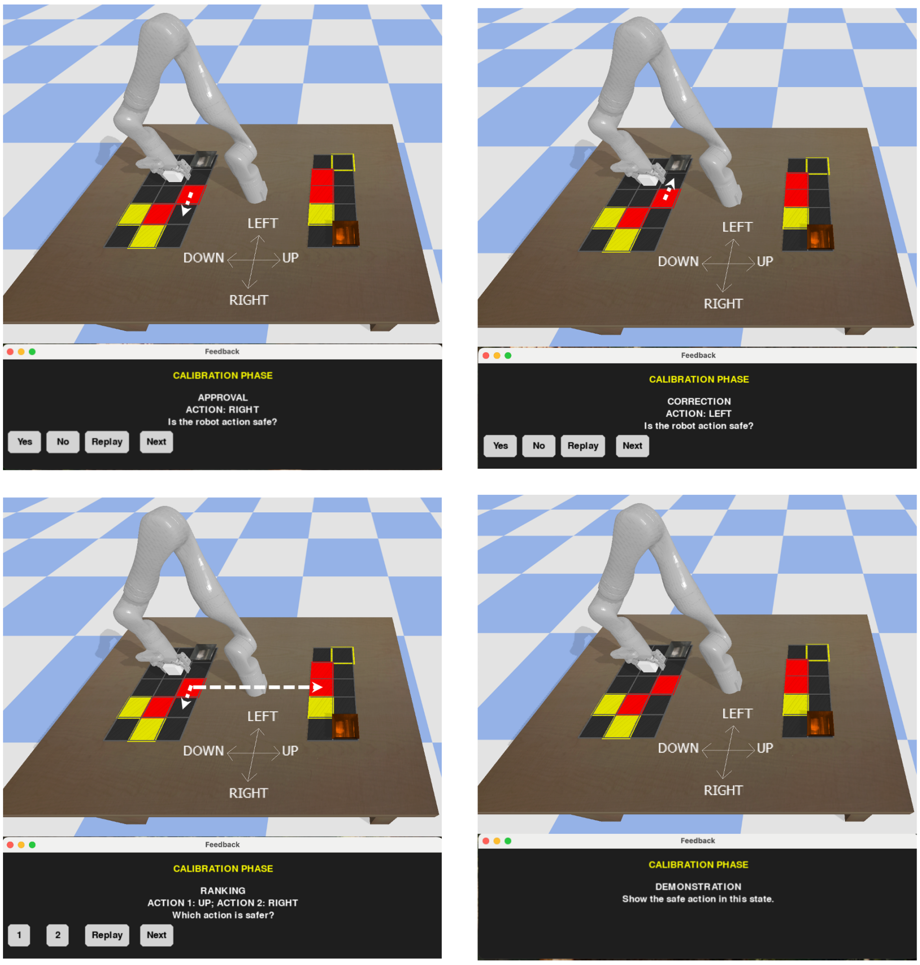

Figure S5: Interface and prompts used in the user study with the Kinova arm. The interface displayed a short clip of the robot's action (indicated by the white dotted arrow) along with a dialog box prompting user feedback. **Top row:** Approval and Correction; **Bottom row:** Ranking and DAM formats.

3.1 User Interface and Feedback Modalities

Figure S5 illustrates the interface and feedback mechanisms used during the in-person user study with the Kinova Gen3 7-DoF robotic arm. Participants interacted with the robot through both a GUI and direct physical manipulation of the arm, depending on the feedback format being queried.

Interface Layout The GUI displayed a simulation of the robot's motion on the tabletop, together with clearly labeled directional arrows (*Up, Down, Left, Right*) corresponding to the robot's possible actions. The lower portion of the interface presented a dialog box, that indicates the current condition, the robot's selected action, and the question or instruction corresponding to the current format. Each query consisted of a brief robot motion in simulation, followed by the corresponding GUI prompt. Participants could replay the motion before submitting their feedback.

REFERENCES

Saran, A., Zhang, R., Short, E. S., and Niekum, S. (2021). Efficiently guiding imitation learning agents with human gaze. In *International Conference on Autonomous Agents and Multiagent Systems (AAMAS)*

Zhang, R., Walshe, C., Liu, Z., Guan, L., Muller, K., Whritner, J., et al. (2020). Atarihead: Atari human eyetracking and demonstration dataset. In *Proceedings of the AAAI Conference on Artificial Intelligence (AAAI)*. vol. 34
